# How do albino fish hear?

**DOI:** 10.1111/j.1469-7998.2010.00762.x

**Published:** 2011-03

**Authors:** W Lechner, F Ladich

**Affiliations:** Department of Behavioural Biology, University of ViennaVienna, Austria

**Keywords:** albinism, pigmentation disorder, hearing impairment, AEP, catfish, *Silurus*, *Corydoras*

## Abstract

Pigmentation disorders such as albinism are occasionally associated with hearing impairments in mammals. Therefore, we wanted to investigate whether such a phenomenon also exists in non-mammalian vertebrates. We measured the hearing abilities of normally pigmented and albinotic specimens of two catfish species, the European wels *Silurus glanis* (Siluridae) and the South American bronze catfish *Corydoras aeneus* (Callichthyidae). The non-invasive auditory evoked potential (AEP) recording technique was utilized to determine hearing thresholds at 10 frequencies from 0.05 to 5 kHz. Neither auditory sensitivity nor shape of AEP waveforms differed between normally pigmented and albinotic specimens at any frequency tested in both species. *Silurus glanis* and *C. aeneus* showed the best hearing between 0.3 and 1 kHz; the lowest thresholds were 78.4 dB at 0.5 kHz in *S. glanis* (pigmented), 75 dB at 1 kHz in *S. glanis* (albinotic), 77.6 dB at 0.5 kHz in *C. aeneus* (pigmented) and 76.9 dB at 1 kHz in *C. aeneus* (albinotic). This study indicates no association between albinism and hearing ability. Perhaps because of the lack of melanin in the fish inner ear, hearing in fishes is less likely to be affected by albinism than in mammals.

## Introduction

Albinism, a genetic abnormality of the melanin system in which the synthesis of this pigment is reduced or lacking, occurs in all classes of vertebrates and in invertebrates. A large diversity of pigmentation disorders is known. Albinism can generally be subclassified as oculocutaneous albinism (four subtypes OCA 1–4 are described) or ocular albinism (OA) depending on loss of melanin in the skin (hair) and eyes versus just in the eyes. Partial albinism describes diseases such as the Waardenburg syndrome, piebaldism and the Tietz–Smith syndrome, in which only part of the skin (hair) lacks pigmentation (Lezirovitz *et al*., 2006). Inherited pigmentary abnormalities occasionally co-occur with hearing impairments in mammals, but no co-occurrence has been reported from other vertebrates. Darwin (1859) has already stated that ‘cats which are entirely white and have blue eyes are generally deaf’. Their colour abnormality and deafness are caused by the dominant white gene *W* (Bergsma & Brown, 1971). Similarly, approximately one-fourth of Dalmatian dogs are at least unilaterally deaf due to genetic disorders (Shelton *et al*., 1993), and spotting mutations in mammals affecting the coat are often associated with hearing impairments (Steel & Bock, 1983).

Several studies have been conducted in rodents such as gerbils, mice, rats and guinea pigs and in cats, leading to quite different results concerning the influence of albinism on hearing. Albino gerbils show significantly lower compound action potentials but equal auditory brain response (ABR) thresholds compared with normally coloured specimens (Szymanski, Henry & Buchting, 1994). In mice, Bartels *et al*. (2001) found no differences in hearing thresholds of normal and albinotic specimens, whereas Cable, Barkway & Steel (1992) stated that albinos with strial melanocytes show evoked potentials of lower amplitude. Significantly lower hearing thresholds have been reported for albinotic guinea pigs by Conlee *et al*. (1986, 1988), whereas Nuttall (1974) found no differences in cochlear microphonic potentials. Albino cats (true *cc* albinos and not dominant white *w*) occasionally show hearing anomalies (Creel, Conlee & Parks, 1983; Conlee *et al*., 1984;).

There are some indications for a relationship, but no general linkage, between OCA and hearing impairments in humans (e.g. Shiloh *et al*., 1990; Amiel *et al*., 1998; Lezirovitz *et al*. 2006;).

Albinism has been reported in numerous fish species, including several catfishes, such as hagfish and lampreys (e.g. Braem & King, 1971), sharks and rays (e.g. Sandoval-Castillo, Mariano-Melendez & Villavicencio-Garayzar, 2006) and numerous bony fishes [i.e. in grunts (Abitia, Aguilar & Aguilar, 1995), or cyprinids (e.g. Ueda, Ishinabe & Jeon, 2007); for a review on albino catfish, see Dingerkus, Seret & Guilbert (1991)]. However, we are not aware of any study investigating the association between albinism and hearing sensitivities in non-mammalian vertebrates including fishes, except for an anecdotal report of a deaf albino goldfish in the early 20th century (Bigelow, 1904).

Popper (1970) showed that Mexican blind cave fish *Astyanax mexicanus* do not differ in hearing sensitivity from the normally pigmented and eyed surface-dwelling populations. Blind cave tetras are OCA albinos according to Protas *et al*. (2006). However, cave-dwelling (hypogean) forms of *A. mexicanus* are not just albinotic forms but differ in many traits from surface-dwelling sighted populations, in particular in their sensory system. They show eye regression, much more developed (lateral line) neuromasts and taste buds.

Dutton *et al*. (2009) mentioned that the inner ears develop abnormally in colourless *Sox10* zebrafish mutants. Mutations of this gene cause Waardenburg syndrome in humans.

In order to determine whether an association between pigmentation disorders (albinism) and auditory sensitivity in fishes exists, we compared the auditory evoked potentials (AEP) and hearing abilities in albinotic and pigmented individuals of two species of fishes possessing hearing specializations. We chose species having excellent hearing sensitivities (often termed hearing specialists) because we hypothesize that slight changes in hearing caused by albinism may affect hearing specialists to a higher degree than species having low hearing sensitivities. Two catfish species, namely the European wels *Silurus glanis* (Siluridae) and the South American bronze catfish *Corydoras aeneus* (Callichthyidae), were studied. These two species represent two different groups of catfishes. *Silurus glanis* has a large free swimbladder and four Weberian ossicles (Weber 1820; Bridge & Haddon, 1893;), whereas *C. aeneus* has paired, tiny, bony, encapsulated swimbladders and a single ossicle (Huysentruyt & Adriaens, 2005; Lechner & Ladich, 2008;). Based on previous studies (Lechner & Ladich, 2008), we expect that the anatomical differences between the two species will be reflected in different auditory sensitivities (at least in normally pigmented specimens) and may be potentially differently affected by albinism. Moreover, the current study is the first to test hearing in the catfish family Siluridae.

## Materials and methods

### Animals

**Figure 1:**
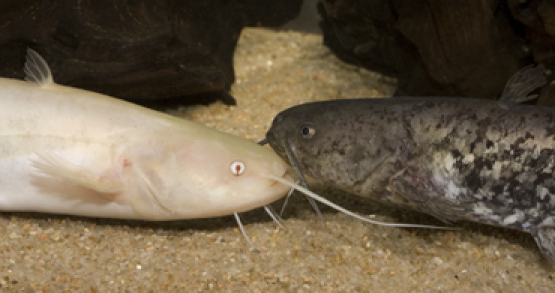
Normally pigmented and albinotic specimens of *Silurus glanis*.

**Figure 2:**
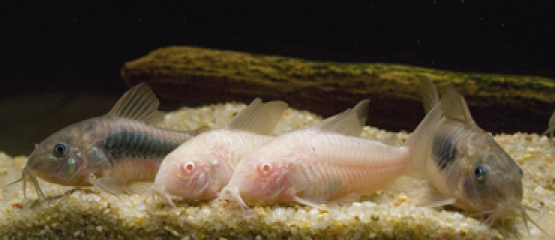
Normally pigmented and albinotic specimens of *Corydoras aeneus*.

Catfishes were obtained from ornamental fish suppliers (*C. aeneus* from Ruinemans Aquarium, Montfoort, the Netherlands, and *S. glanis* albinos from Hornbach, Brunn am Gebirge, Austria) and a fish hatchery (*S. glanis* normal from Fischzucht Pottenbrunn, Pottenbrunn, Austria) (Figs 1 and 2).

Fish were kept in planted aquaria with a sand bottom equipped with roots and clay or plastic tubes as shelters. Only external filters were used in order to provide a quiet environment. The temperature was maintained at 20±1 °C for *S. glanis* and 25±1 °C for *C. aeneus*. A 12 h:12 h L:D cycle was maintained. *Silurus glanis* were fed frozen food (chironomid larvae, smelt *Osmerus* sp., *Gammarus* sp. and turkey heart) and live earthworms, *C. aeneus* were fed frozen chironomid larvae and artificial food (granulate, flakes and tablets). Standard length (SL) was measured as ‘standard length 2’ following Holcik, Banarescu & Evans (1989): *Silurus* specimens ranged from 152 mm SL and 35 g body weight to 215 mm SL and 85 g, *Corydoras* specimens ranged from 32.7 mm SL and 1.53 g to 45.7 mm SL and 3.34 g.

### Auditory sensitivity measurements

Hearing thresholds were obtained using the AEP recording technique developed by Kenyon, Ladich & Yan (1998), with slight modifications. The exact procedure, including the presentation of sound stimuli and statistical analysis is described by Lechner, Wysocki & Ladich (2010).

Hearing thresholds were determined at 0.05, 0.07, 0.1, 0.3, 0.5, 0.8, 1, 2, 3, 4 and 5 kHz. Test subjects were mildly immobilized with Flaxedil (gallamine triethiodide; Sigma-Aldrich, Vienna, Austria) diluted in a Ringer solution. The dosage applied was 2.12–3.39 *μ*g g^−1^ for *S. glanis* normal, 3.57–5.78 *μ*g g^−1^ for *S. glanis* albino, 0.41–0.62 *μ*g g^−1^ for *C. aeneus* normal and 0.55–0.85 *μ*g g^−1^ for *C. aeneus* albino. Water temperature during recordings was adjusted to 20±1 °C for *S. glanis* and 25±1 °C for *C. aeneus*.

## Results

### Pigmentation

Albino specimens of both species showed uniform body coloration from whitish pink in the ventral to yellowish orange in the dorsal region and the fins, combined with red eyes (Figs 1 and 2). Pigmented *S. glanis* had black eyes and a high variability in body pigmentation. Some individuals were dark brown with numerous black and only few silvery whitish blotches and dots, whereas in other specimens, the number of whitish blotches was much higher, covering most of the brown body (Fig. 1). Normal *C. aeneus* were uniformly coloured (black eyes and a yellowish-brown to delicate reddish-brown body colour with strong metallic glint on the sides and darker regions in the middle of the flanks) (Fig. 2). All catfish were acclimated to our aquaria for at least 3 weeks before testing.

### AEP waveforms

**Figure 3:**
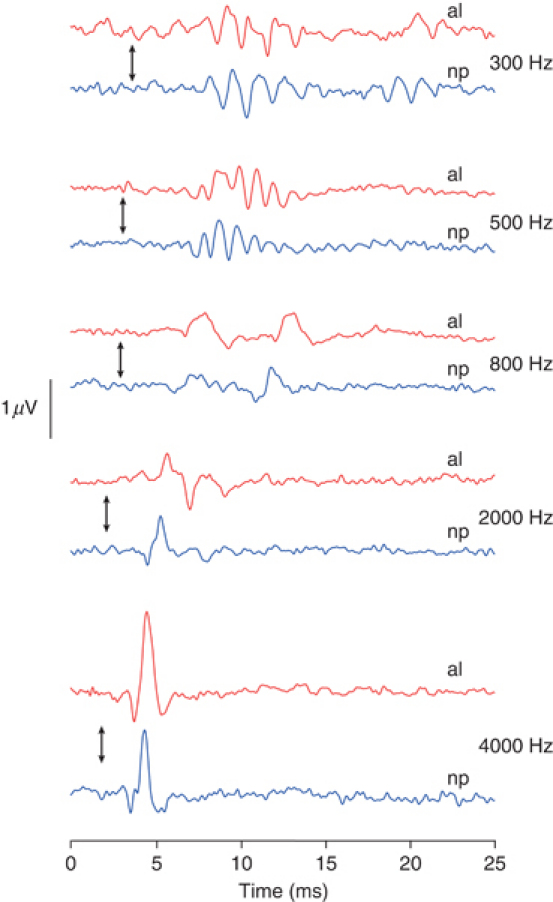
Waveforms of auditory evoked potentials at representative frequencies in normally pigmented (np) and albinotic (al) *Silurus glanis*. Arrows indicate stimulus onset.

**Figure 4:**
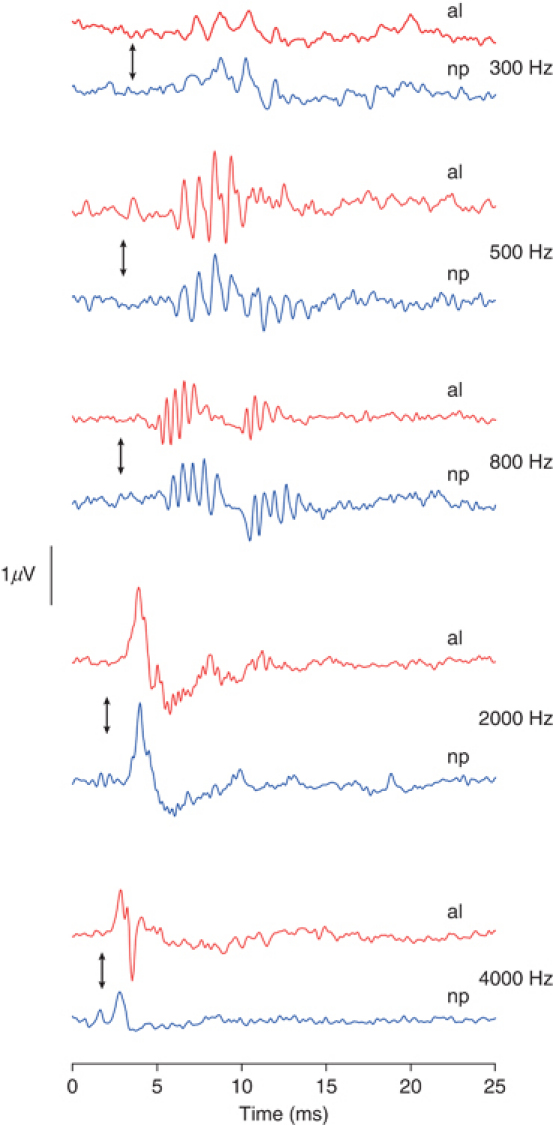
Waveforms of auditory evoked potentials at representative frequencies in normally pigmented (np) and albinotic (al) *Corydoras aeneus*. Arrows indicate stimulus onset.

The AEP of pigmented and albinotic groups of each species did not differ in the overall shape of waveforms, latency, amplitudes or in the number of positive or negative peaks (Figs 3 and 4).

### Auditory thresholds

**Figure 5:**
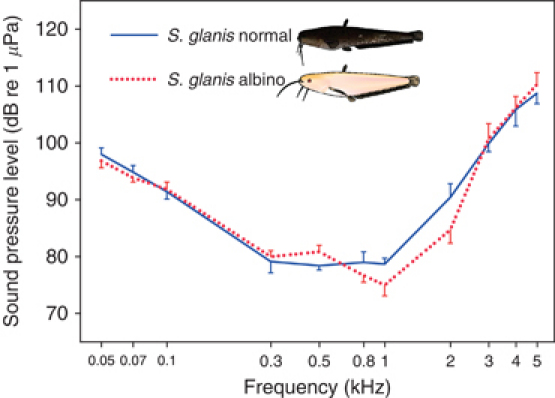
Mean (±sem) hearing thresholds of normal and albino *Silurus glanis*.

Hearing thresholds of *S. glanis* were the lowest between 300 and 1000 Hz; the lowest threshold was 78.4 dB at 500 Hz in pigmented *S. glanis* and 75 dB at 1000 Hz in albinos (Fig. 5, Table 1). Comparison of the audiograms of the two groups by a two-way ANOVA revealed no significant overall differences (*F*_1,132_=0.854, *P*=0.357). Comparison of hearing thresholds at each frequency with an unpaired *t*-test showed no significant differences between normally coloured and albino fish (*P*>0.05).

**Table 1:** Mean hearing thresholds (±sem) of the normal and the albinotic specimens in the two species studied

	Hearing thresholds (dB re 1 *μ*Pa)			
	
Frequency (kHz)	*Silurus glanis*	*Silurus glanis* albino	*Corydoras aeneus*	*Corydoras aeneus* albino
0.05	98.00 ± 1.11	96.86 ± 1.22	91.57 ± 1.29	91.00 ± 1.35
0.07	94.86 ± 1.16	93.86 ± 0.74	87.29 ± 1.76	86.71 ± 1.86
0.1	91.43 ± 1.29	91.86 ± 1.24	84.86 ± 0.99	85.71 ± 1.21
0.3	79.14 ± 1.97	80.00 ± 1.05	79.43 ± 1.27	79.29 ± 1.69
0.5	78.43 ± 0.75	80.86 ± 1.14	77.57 ± 1.85	78.00 ± 1.22
0.8	79.00 ± 1.86	76.71 ± 1.23	80.29 ± 1.23	78.43 ± 2.00
1	78.71 ± 1.02	75.00 ± 1.91	80.57 ± 1.00	76.86 ± 1.81
2	90.43 ± 2.39	84.71 ± 2.37	101.86 ± 1.14	103.71 ± 1.55
3	99.86 ± 1.39	100.71 ± 2.64	109.43 ± 0.97	108.71 ± 0.87
4	105.86 ± 2.87	106.14 ± 2.05	112.57 ± 1.04	111.14 ± 1.08
5	108.71 ± 1.81	110.29 ± 2.08	113.43 ± 1.41	114.29 ± 1.06

**Figure 6:**
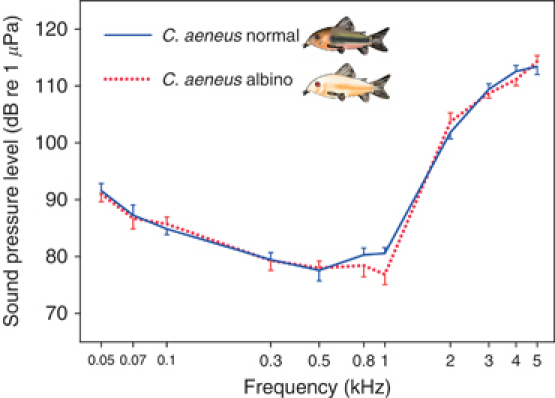
Mean (±sem) hearing thresholds of normal and albino *Corydoras aeneus*.

In *C. aeneus*, auditory sensitivity was the highest between 300 and 1000 Hz. The maximum sensitivity was 77.6 dB at 500 Hz in pigmented and 76.9 dB at 1000 Hz in albinotic *C. aeneus* (Fig. 6, Table 1). Comparing the audiograms of the two groups by a two-way ANOVA revealed no significant overall differences (*F*_1,132_=0.590, *P*=0.444). Comparing hearing thresholds at each frequency with an unpaired *t*-test showed no significant differences between the normally pigmented and the albino group (*P*>0.05).

## Discussion

### AEP waveforms

AEP waveforms of the studied catfish species did not differ between normal and albinotic specimens of each species (Figs 3 and 4). This is in agreement with Dum, Schmidt & von Wedel (1980), who found no difference in the latency and amplitude of the individual peaks in the brain stem response of normal and albinotic guinea pigs. In contrast, Cable *et al*. (1992) reported that *W*^*v*^*/W*^*v*^ mouse mutants showed lower or no endocochlear potentials. Significant differences have been observed in the ABRs of normal and albinotic humans (Creel *et al*., 1980) and between normal and albinotic (real *cc* and not *W*, which is associated with deafness) cats (Creel *et al*., 1983). This is most likely due to differences in auditory pathways and projections between nuclei in albino and pigmented animals (Moore & Kowalchuk, 1988). In general, the AEP waveforms in our catfish were in accordance with those in previous studies in fishes (e.g. Kenyon *et al*., 1998; Ladich, 1999; Wysocki & Ladich, 2001;). Latency and duration of responses decreased with increasing frequencies.

### Hearing sensitivity

Hearing impairment and pigmentation disorders frequently occur together in many syndromes in mammals (Lezirovitz *et al*., 2006). The exact mechanism has been discussed for many years (Garber *et al*., 1982; Barrenas & Axelsson, 1992;). The area primarily involved in many of the pigmentation disorders is the stria vascularis of the mammalian cochlea. Melanocytes within the stria vascularis generate the endocochlear potential (Cable *et al*., 1992; Bartels *et al*., 2001;), and the absence or degeneration of melanocytes is assumed to be responsible for hearing abnormalities in albino mammals.

As noted above, comparative studies on hearing abilities in normally pigmented and albinotic specimens revealed widely differing results in AEPs and hearing thresholds. A main reason for these inconsistencies may be the term ‘albinism’, which is much too sketchy to describe the heterogeneous reasons for defects in pigment production in vertebrates.

Besides the general differentiation between OA and OCA (Orlow, 1997), there are several subclassifications of these two types of albinism, and further pathological syndromes expressed as hypopigmentation (among other effects) are known.

OCA is a group of four autosomal recessive disorders (OCA1–OCA4) caused by either a complete lack or a reduction of melanin biosynthesis in the melanocytes, resulting in hypopigmentation of the hair, skin and eyes (Gronskov, Ek & Brondum-Nielsen, 2007). Studies on hearing in albino vertebrates normally use specimens with some type of OCA, but most studies do not name the type of albinism of the experimental animals. Our albino catfish show OCA2, as did the Mexican cave tetras (Protas *et al*., 2006; Jeffery, 2009;).

In fish that lack a cochlea and a stria vascularis, melanocytes – in particular their absence in the inner ear – may play a less crucial role than in the mammalian ear. This may explain why our study animals did not exhibit pigmentation-related differences in hearing abilities. Nonetheless, hypopigmentation could potentially co-occur with hearing disorders in zebrafish mutants. Dutton *et al*. (2009) showed that knocking out the *Sox10* gene results in abnormalities in ear development and lack of body pigmentation, whereas the eyes remain normally pigmented.

Fish lacking pigmentation have been tested for hearing by Popper (1970) only. As in our study, hearing abilities of two closely related species, the Mexican blind cave fish *Astyanax jordani* and its eyed ancestor *A. mexicanus*, did not differ. Today, *A. jordani* is not regarded as a valid species, but as a blind, depigmented, cave-dwelling form of *A. mexicanus* (Jeffery, 2009). In fact, several cave populations evolved convergently from surface-dwelling populations of *A. mexicanus* (Jeffery, 2006) in different habitats. Contrary to the albino catfish of the current study in which lack of pigmentation is based on a genetic disorder, blind cave fish are adapted to cave dwelling and differ in several traits other than pigmentation from the surface-dwelling, pigmented populations. They exhibit major changes in their sensory system, including eye regression, larger lateral line neuromasts and modified taste buds (Bleckmann, 1993; Boudriot & Reutter, 2001; Montgomery, Coombs & Baker, 2001;). According to Teyke (1990), the cupulae of neuromasts are about twice as long as in sighted populations, which (most likely) results in higher sensitivity of neuromasts and thus better orientation in caves. Boudriot & Reutter (2001) mentioned that taste buds in cave-dwelling fish contain significantly more axons than in sighted river fish, which indicates a compensatory improvement of chemoreception for prey detection.

In summary, based on our current results, we assume that albinism is not associated with hearing disorders in fishes.

### Hearing in catfishes

The order of catfishes comprises more than 3000 species (Ferraris, 2007). Catfishes belong to otophysines and possess Weberian ossicles, which connect the swimbladder to the inner ear. Hearing abilities of many siluriform species are among the best in fishes, with regard to both the detectable frequency range and the absolute sensitivity. The two species chosen represent two different groups of catfishes. *Siluris glanis* has one large free swimbladder and four Weberian ossicles, whereas *C. aeneus* has paired, tiny, bony, encapsulated swimbladders and a single ossicle, which should result in better high-frequency hearing in *S. glanis* (Lechner & Ladich, 2008). While this is the case, the hearing acuity of *S. glanis* is quite low at high frequencies compared with other species with a well-developed swimbladder and ossicles. At 3 kHz and at higher frequencies, *S. glanis* exhibited higher hearing thresholds than all but one catfish species with free bladder tested so far, which belonged to the families Ariidae, Auchenipteridae, Heptapteridae, Malapteruridae, Mochokidae and Pseudopimelodidae (Lechner & Ladich, 2008; Lechner *et al*., 2010;), Doradidae (Ladich, 1999), Ictaluridae (Poggendorf, 1952; Wysocki, Montey & Popper, 2009;) and Pimelodidae (Ladich, 1999; Wysocki *et al*., 2009;).

As expected, the hearing abilities of the callichthyid *C. aeneus* are similar to its congenerics *Corydoras paleatus* and *Corydoras sodalis* (Ladich, 1999; Lechner & Ladich, 2008;), with *C. paleatus* having a lower high-frequency hearing sensitivity than the two other species.

In summary, the hearing abilities of the silurid *S. glanis* and the callichthyid *C. aeneus* fit the concept that hearing abilities in catfishes depend on the swimbladder size and the number of Weberian ossicles, as stated by Lechner & Ladich (2008). Albinism does not affect hearing in either of these two species.
